# Wildlife fertility control: a mini-review of technologies, efficacy, and welfare impacts

**DOI:** 10.1590/1984-3143-AR2026-0114

**Published:** 2026-10-09

**Authors:** Jens Jacob, Giovanna Massei

**Affiliations:** 1 Botstiber Institute for Wildlife Fertility Control, Media, PA, United States; 2 Julius Kühn-Institute – JKI, Federal Research Institute for Cultivated Plants, Institute for Epidemiology and Pathogen Diagnostics, Rodent Research, Münster, Germany; 3 Department of Environment and Geography, University of York, York, United Kingdom

**Keywords:** contraception, human-wildlife-conflict, population management, vertebrate management

## Abstract

This review examines the growing challenge of managing overabundant wildlife populations in the context of increasing human–wildlife conflicts. Traditionally, population control has relied on lethal methods such as culling, but these approaches are increasingly restricted due to concerns over environmental harm, non-target effects, human safety, and shifting societal values favouring more humane solutions. Fertility control has emerged as a promising non-lethal alternative, aiming to regulate populations by reducing reproductive success rather than increasing mortality. Its effectiveness depends on species-specific life-history traits: it can rapidly reduce populations of short-lived, highly fecund species, while effects in long-lived animals are delayed. Various fertility control methods are available, including endocrine suppression, immunocontraceptive vaccines, synthetic hormones and non-endocrine approaches such as zona pellucida vaccines. A major limitation to field applications of fertility control is the development of efficient delivery systems, particularly for small or free-ranging species where oral delivery must overcome challenges of palatability, stability, and species-specificity. Animal welfare considerations depend on species, contraceptives and contexts and involve trade-offs between reduced mortality and potential impacts on behaviour, physiology, and reproduction. Nevertheless, fertility control may offer welfare advantages over lethal methods and can reduce the transmission of infectious agents by maintaining social stability. Despite its potential, widespread implementation is constrained by economic and regulatory barriers. Overall, fertility control represents a valuable tool within integrated wildlife management strategies, though continued technological innovation and regulatory adaptation are essential for widespread application in the future.

## Introduction

The management of overabundant wildlife populations is a global challenge as human development continues to encroach upon natural habitats, leading to escalating environmental, economic, and health issues as well as social impacts (Abrahms et al., 2023). It is relevant for species as small as house mice (*Mus musculus*) and as large as African elephants (*Loxodonta africana*). Overabundant species, whether native or introduced, can cause significant crop losses, infrastructure damage and the transmission of infectious agents (pathogens and parasites) to livestock and humans (Barfield et al., 2006). The combination of plant, animal, human and environmental health puts some wildlife taxa such as rodents in the centre of the One Health concept. Traditional population management has long relied on lethal techniques such as culling (trapping, hunting, use of poison). However, these methods are increasingly restricted or banned in various jurisdictions around the world due to concerns about their impact on the environment in general and non-target impact in particular, human safety and a general shift in public values towards more humane treatment of animals. These trends are expected to intensify in the future (Jacob and Singleton, 2026) due to global expansion of human-wildlife overlap (Ma et al., 2024).

Human-wildlife conflict leads to negative impact on humans (safety, infrastructure, livelihoods, health) or to resulting human action that may harm animals or lead to population decline in wildlife. Fertility control has emerged as a prominent alternative to lethal control to minimise human-wildlife conflict, viewed by the public as a more humane and socially acceptable tool for mitigating human-wildlife conflict (Massei, 2023). The potential to manage populations by manipulating reproductive success rather than increasing mortality rates has been researched for over five decades. While earlier efforts faced hurdles due to a lack of understanding regarding species-specific reproductive strategies (Barfield et al., 2006), rapid technological advancements in immunocontraception, synthetic steroids, and genetic biocontrol are providing new management options in the more or less distant future (Hinds et al., 2026). This review examines the current status of wildlife fertility control agents, their mechanisms of action, biological feasibility across different taxa, and the complex animal welfare and regulatory considerations that affect their practical use.

## Biological feasibility and population dynamics

The effectiveness of fertility control as a management tool is fundamentally linked to the life-history traits of the target species (Dolbeer, 1998). Population models indicate that reproductive control can be efficient for managing short-lived, highly fecund species such as small birds (Dolbeer, 1998) or rodents where population outbreaks could be dampened (Davis et al., 2003; Jacoblinnert et al., 2022). For these species, high natural mortality and rapid population turnover mean that reducing recruitment can lead to significant population declines within a relatively short timeframe. In contrast, for long-lived species with low reproductive rates, such as deer, horses (*Equus caballus*), and elephants, fertility control exerts its effect on population size after several years (Fagerstone et al., 2002; Massei, 2023). Because infertile individuals remain in the population for their natural lifespan, culling remains relevant when immediate population reduction is the aim such as in commensal rodents in human housing or in food industry facilities. Other models based on long-lived but fast reproductive species such as wild boar (*Sus scrofa*) suggest that integrating fertility control with culling is more efficient or faster than culling alone (Croft et al., 2020; Pepin et al., 2017).

The proportion of the population that need to be rendered infertile to achieve management goals varies significantly. For eruptive species like house mice, models suggest that 50-80% of females should be treated to achieve a population reduction (Davis et al., 2003; Jacoblinnert et al., 2022). Social systems and mating strategies also play a role; in polygamous species, targeting females is generally more efficient, as rendering a large proportion of males infertile may not significantly impact the number of births if the remaining fertile males can still mate with most females (Bomford, 1990; Fagerstone et al., 2002).

## Mechanisms of action and current agents

The reproductive system of vertebrates can be targeted through various mechanisms that either temporarily or permanently disrupt fertility.

**Endocrine suppression** usually works through disrupting the hypothalamic-pituitary-gonadal (HPG) axis, which regulates reproduction in all mammals. Gonadotropin-releasing hormone (GnRH) is targeted directly as a master regulator. Its suppression prevents the release of Luteinising Hormone (LH) and Follicle Stimulating Hormone (FSH), thereby inhibiting ovulation and sperm production. GnRH agonists, like deslorelin acetate have been used as long-acting implants in carnivores (Trigg et al., 2001), macropodids (Herbert et al., 2005), and ungulates (Patton et al., 2007).

**Immunocontraceptive vaccines** induce an immune response against essential reproductive hormones or proteins. GonaCon™, an anti-GnRH vaccine, causes infertility in both sexes and has been registered for use in white tailed deer (*Odocoileus virginianus*), horses, and prairie dogs in the USA. In addition, it has also shown multi-year efficacy in wild boar, and feral cattle (*Bos taurus*) (Massei et al., 2012; Pinkham et al., 2022). Combinations of synthetic hormones such as oestrogens and progestins in the product EP-1™ (quinestrol and levonorgestrel) have been widely tested in several rodent species (reviewed in Jacoblinnert et al., 2022). These steroids disrupt the oestrous cycle and reduce litter sizes, often with reversible effects. In larger mammals, such as kangaroos and koalas (*Phascolarctos cinereus*), levonorgestrel implants provide long-term infertility (Nave et al., 2002).

**Physical and chemical non-endocrine methods** prevent fertilisation or implantation without necessarily altering reproductive hormone levels. Zona pellucida immunocontraception uses Porcine zona pellucida (PZP) vaccines that stimulate the production of antibodies that prevent the sperm to fertilize the egg. PZP is highly effective in horses, deer, and elephants but is ineffective in rodents (Kirkpatrick et al., 2011). Formulations like PZP-22 and SpayVac™ aim for multi-year efficacy from a single dose in large mammals. Chemosterilants such 4-vinylcyclohexene diepoxide (VCD) cause the depletion of follicles in female ovaries, potentially leading to permanent sterility. However, VCD has to be delivered continuously for a long period (Mayer et al., 2002). ContraPest™, which combines VCD and plant derived triptolide, is registered for the management brown rats (*Rattus norvegicus*) and roof rats (*Rattus rattus*) in the USA. Plant based compounds like triptolide (from the thunder god vine (*Tripterygium wilfordii*)) and neem (*Azadirachta indica*) extracts have been researched for their ability to impair spermatogenesis or disrupt the oestrous cycle. While promising, many plant extracts require continuous consumption and can be unpalatable at high doses. Some plant-based products are commercially available in the USA but there is no published information about efficacy, safety and side effects on targets and non-target species.

**Genetic biocontrol** is an emerging technology without registered products available. CRISPR/Cas9-mediated gene drives aim to rapidly spread fertility-inhibiting mutations through a population. While still largely in the laboratory phase for mammals, "t-CRISPR" approaches in house mice leverage natural selfish genetic elements to increase inheritance bias toward sterile phenotypes (Gierus et al., 2022; Runge and Lindholm, 2018).

## Delivery methods

A major hurdle for large-scale wildlife fertility control is the development of cost-effective delivery systems. The smaller the target species, the less suitable are approaches that focus on delivering contraceptives to individuals, which is the main strategy when using injectable contraceptive vaccines in large species such as African elephants, feral horses, wild boar and white-tailed deer.

Most wildlife contraceptives are available as injectables, requiring individual capture or remote delivery via darting. While remote darting is feasible for small, habituated populations, it remains resource-intensive for large-scale management (Nielsen et al., 1997). Orally deliverable agents are essential for managing rodents and other small and wide-ranging species. The challenges in formulating orally delivered contraceptives include palatability, maintain effectiveness of the contraceptive through the gastrointestinal tract and stability of the active ingredient under varying environmental conditions (Sharma and Hinds, 2012). Furthermore, baits must be delivered to a sufficient proportion of the target population for fertility control to have an effect on population growth rate. Most current agents are not species-specific. Specificity is often achieved through tailored delivery systems, such as species-specific bait stations (e.g., the Boar Operated System (Massei et al., 2010). Restricting access to bait is highly relevant for human safety if compounds can affect humans. For the use of anti-fertility bait in rodent management, much can be drawn from the existing risk mitigation measures used to avoid exposure of humans and non-target wildlife to rodenticidal bait. AI-aided systems (Chen et al., 2019) developed for individual species recognition may help in the future to deliver bait to targets only (Charlton et al., 2024).

## Animal welfare and ethical considerations

The humaneness of fertility control is a complex issue involving short-term stress in relation to contraceptive administration, physiological changes and the potential for long-term behavioural impacts (Hampton et al., 2015). Administration procedures requiring animal capture, restraint and surgery impose significant welfare costs and remote darting can cause injuries. Immunocontraceptives cause injection site reactions such as sterile abscesses in some species (deer, horses, e.g. Gionfriddo et al., 2011) but not in others (wild boar, e.g. Massei et al., 2012).

Contraceptives may alter natural behaviour. For example, treated animals may lose dominance (Jacob and Matulessy, 2004) or natural behaviour and secondary sexual characteristics (Bertschinger et al., 2001). Non-endocrine methods like PZP allow animals to remain hormonally competent and in feral horses have been associated with increased body condition and longevity with no significant changes in social organization or behaviours (Kirkpatrick et al., 2011). However, in other species, like white-tailed deer treated with a PZP-based vaccine and possums treated with tubal ligation, fertility control leads to extended breeding seasons as females fail to conceive and repeatedly return to oestrus (Ji et al., 2000; Miller et al., 1999). This extension can increase energetic costs and social perturbation, particularly in males (Ji et al., 2000). Modern welfare science emphasises the importance of allowing animals to express natural behaviours and experience positive affective states. Depriving nurturing species of the opportunity to express parental behaviour however is an unavoidable welfare cost of all fertility control modalities (Hampton et al., 2015). Conversely, pregnancy, lactation and rearing offspring have considerable physiological and energy cost (Speakman, 2008) that are alleviated by fertility control which may improve the wellbeing of animals. Density-dependent benefits, by reducing population density, can alleviate resource competition, and risk of pathogen transmission, potentially improving the body condition and survival of both treated and untreated individuals. Reduced competition for food and space can lead to a net increase in the average welfare of a population (Eckerström Liedholm et al., 2024). In comparison to lethal approaches (hunting, trapping, using poisons) it seems obvious that effective fertility control should have a clearly better welfare performance because there is no killing and suffering related to the symptoms of lethal (chemical) methods.

In the context of wildlife and zoonotic diseases, the application of fertility control has several advantages over culling. In many species, culling disrupts social organisation and increases animal movements, thus leading to increased contact rates and risks of transmission of infectious agents (e.g. Choisy and Rohani, 2006). Conversely, fertility control is less likely to cause social perturbation than culling (Riordan et al., 2011).

## Economic and regulatory hurdles

The registration of wildlife contraceptives is constrained by high development costs; in parallel, for rodent management there is a large market currently dominated by rodenticides. The dossier for registration of novel contraceptives, which varies in different countries, requires extensive data on safety and effectiveness, as well as an assessment of risks to humans and the environment. For instance, in some countries, wildlife contraceptives are regulated as veterinary medicines, requiring the same stringent safety, efficacy, and environmental assessments as drugs for domestic animals. This creates a mismatch because these products are intended for free‑living wildlife, not managed livestock. In addition, regulators require extensive data on environmental fate, non‑target species exposure, and ecosystem impacts. For wildlife contraceptives, especially oral or remotely delivered ones, collecting such data is difficult and expensive. Together, these barriers mean that very few wildlife contraceptives are currently registered, despite growing need for non‑lethal population control in urban areas, national parks, and human‑wildlife conflict zones.

## Conclusions

Wildlife fertility control has transitioned from a theoretical concept to a rapidly advancing field of practical management. While it is not a panacea – especially where immediate population reduction is required – it offers a vital, non-lethal tool for the integrated management of overabundant wildlife with the potential to minimise the effect of overabundant wildlife on human, animal and environmental health (One Health) (Rhyan et al., 2013), crops, infrastructure and in other sectors. The success of future applications will depend on several key factors: the development of effective oral delivery systems for small and cryptic species; the identification of more species-specific agents; and a deeper understanding of the long-term behavioural and welfare implications of various modalities that must then be compared to traditional methods of wildlife management.

Investing in these technologies is crucial for regions where high human population densities and shifting social values demand sustainable methods for promoting human-wildlife coexistence. By integrating fertility control with traditional management and addressing the remaining technological and regulatory hurdles, wildlife managers can better mitigate conflicts while upholding the animal welfare standards increasingly demanded by society.

## Data Availability

No research data was used.
